# Psychiatric Manifestations of Delta-8-Tetrahydrocannabinol (Δ8-THC) Use Among Patients With Preexisting Psychiatric Disorders: A Case Series

**DOI:** 10.7759/cureus.79621

**Published:** 2025-02-25

**Authors:** Harmit Singh, Rohini Garg, Rajesh Tampi

**Affiliations:** 1 Psychiatry, Creighton University School of Medicine, Omaha, USA; 2 Internal Medicine, CHI Health Mercy Hospital, Council Bluffs, USA

**Keywords:** cannabis, delta-8, psychiatric comorbidity, psychosis, thc

## Abstract

Delta-8-tetrahydrocannabinol (Δ8-THC) products, made from hemp-derived cannabidiol, have become more widely available since changes in legislation allowed hemp to be excluded from controlled substances. These products are often perceived as a safe, legal, and accessible alternative to marijuana or delta-9-tetrahydrocannabinol. However, scientific research on their effects remains limited. There have been reports of psychiatric symptoms associated with Δ8-THC use, although the literature is not yet comprehensive. In this report, we present three cases of patients with distinct preexisting psychiatric disorders who developed various psychiatric manifestations, including psychosis, after using Δ8-THC products. All three individuals had purchased these products legally, believing them to be safe and effective for managing their symptoms. However, their use ultimately led to psychiatric symptoms severe enough to require hospitalization. This case series highlights the need for greater awareness of the potential psychiatric effects of Δ8-THC, particularly among individuals with preexisting mental health conditions, and underscores the critical gap in research on these products.

## Introduction

The passage of the Agricultural Improvement Act of 2018, also known as the Farm Bill, removed hemp from the list of controlled substances [1]. Under this legislation, cannabis plants containing less than 0.3% delta-9-tetrahydrocannabinol (Δ9-THC) and their derivatives were no longer classified as scheduled substances [1]. As a result, the production and legal sale of noncontrolled psychoactive delta-8-tetrahydrocannabinol (Δ8-THC) products, synthesized from hemp-derived cannabidiol (CBD), increased significantly. These products have gained popularity due to their legal accessibility and public perception as safer alternatives to marijuana or Δ9-THC.

Δ8-THC is an isomer of Δ9-THC and has a nearly identical chemical structure to Δ9-THC, the primary psychoactive compound in cannabis [2]. Like Δ9-THC, Δ8-THC acts on cannabinoid receptors in the brain [2]. Extensive research has established a correlation between Δ9-THC use and psychosis [3-5], raising concerns that Δ8-THC may produce similar clinical effects [6]. However, there is currently no focused research on the long-term effects of Δ8-THC.

This case series highlights psychiatric manifestations observed in three patients with distinct preexisting psychiatric disorders who developed various symptoms, including psychosis, after consuming Δ8-THC products. All three individuals had obtained these products legally, believing them to be safer alternatives to marijuana. Their experiences underscore the need for greater awareness of the potential psychotic effects of Δ8-THC, particularly in individuals with preexisting mental health conditions. Additionally, this case series emphasizes the urgent need for research on Δ8-THC products, which have become increasingly popular due to their widespread availability and legal status [7].

## Case presentation

Case 1

A 32-year-old female with a psychiatric history of post-traumatic stress disorder (PTSD), borderline personality disorder, self-harming behaviors, drug overdose, and depression presented to the emergency department with heightened paranoia, delusions, and auditory hallucinations. She reported hearing her neighbors’ voices through the speakers on her floors and expressed concerns that they were tampering with her belongings and intending to harm her. Her extreme paranoia and discouraging voices prevented her from seeking help. She complained of constant suicidal ideation and admitted to contemplating jumping from a bridge.

In the emergency room, she received olanzapine 10 mg and lorazepam 3 mg to manage her symptoms. On admission, her vital signs included a blood pressure of 189/106 mmHg, a heart rate of 114 bpm, a respiratory rate of 30 breaths per minute, and a temperature of 97.5°F. Laboratory workup, including a complete blood count, comprehensive metabolic panel, thyroid-stimulating hormone, and free T4 levels, was unremarkable. A urine drug screen was positive for cannabinoids. Her home medications included propranolol 10 mg twice daily, sumatriptan 50 mg as needed for headaches, lorazepam 0.5 mg as needed for panic attacks, and buspirone 15 mg twice daily. She was diagnosed with psychosis unspecified and PTSD.

On admission, she displayed disorganized behavior, increased psychomotor activity, anxiety, and restlessness. She appeared anxious and paranoid, frequently scanning her environment, with a labile and tearful affect. Her thought content included paranoid and delusional ideation but no homicidal or suicidal plans. During hospitalization, she was started on risperidone for psychotic symptoms, clonidine for blood pressure management, and trazodone for sleep and PTSD-related nightmares. The risperidone dose was titrated up to 1.5 mg twice daily, and she responded well to treatment.

During her hospitalization, she reported that her mental health had deteriorated over the past year, coinciding with the escalating use of Δ8-THC gummies while working at a store selling Δ8-THC products derived from hemp-based CBD. As her consumption increased, she experienced worsening paranoia, disrupted sleep, and auditory hallucinations. She eventually quit her job. Despite prior use of illicit substances, including marijuana since the age of 17, she had not previously experienced psychotic episodes.

After 13 days in the hospital, she returned to her baseline functioning. She was discharged on risperidone 1.5 mg twice daily, clonidine 0.2 mg three times daily, trazodone 300 mg at bedtime, and hydroxyzine 25 mg as needed for anxiety up to three times daily. She was advised to abstain from all cannabis products, including marijuana and Δ8-THC. At her outpatient psychiatric follow-up, her psychotic symptoms had resolved, and she continued to respond well to treatment.

Case 2

A 27-year-old male with a psychiatric history of substance (cannabis)-induced psychosis, hallucinations, self-harming behaviors, and depression, as well as a prior psychiatric admission a year ago, presented to the emergency department with disorganized behavior and confusion. He was accompanied by his cousin, who was concerned after his unannounced visit from another city and multiple encounters with the police due to car-related issues. He had recently been fired from his job for repeated absences.

His cousin suspected he had relapsed into cannabis use, as his bizarre behavior resembled the psychotic episode that led to his hospitalization the previous year. The patient described experiencing “neurosis” and increased stress at work. He reported poor sleep and stated that he did not remember how he had made it to her house. While he denied hallucinations, he exhibited confusion and a detached demeanor during the assessment. An admitting diagnosis of psychosis unspecified was made, with a differential diagnosis of substance-induced psychosis.

His prior hospitalization had been due to cannabis-induced psychosis, for which he was treated with olanzapine and advised to discontinue cannabis use. He had successfully stopped using cannabis and had been making progress in his life. However, in recent months, he began using legally procured Δ8-THC gummies from a CBD store to manage anxiety and depression, believing them to be a safe and legal alternative to marijuana.

On admission, his vital signs included a blood pressure of 140/90 mmHg, a heart rate of 106 bpm, a respiratory rate of 16 breaths per minute, and a temperature of 36.5°C (97.7°F). A urine drug screen was positive for cannabinoids. He admitted to consuming a delta-8 edible the night before admission. He was dressed appropriately but exhibited slowed psychomotor activity, poor eye contact, and a flat affect. His thought process was disorganized, with difficulty focusing and concentrating. He appeared lost in thought and showed signs of responding to internal stimuli.

He was admitted to the psychiatric unit for stabilization. Initially, he remained isolated in his room with a flat affect. He was started on fluphenazine 2 mg three times daily, in addition to his home dose of bupropion for depression. Trazodone and propranolol were prescribed for sleep and anxiety. After 11 days in the inpatient psychiatric unit, he returned to his baseline functioning. He was discharged on fluphenazine decanoate 12.5 mg intramuscular injection every 14 days, along with oral fluphenazine 1 mg twice daily. He returned to his hometown but was subsequently lost to follow-up.

Case 3

A 21-year-old male with no prior psychiatric history or hospitalizations was brought to the emergency department by his aunt due to bizarre behavior and grandiose delusions. His family reported that he believed he could fly and was found on a bridge, expressing a desire to jump off to safety. He had been reported missing a few days earlier and was later found swimming in a river. He claimed to have been assaulted and presented with multiple abrasions. Upon evaluation, he exhibited rapid speech, flight of ideas, and a tendency to relate his experiences to Buddhism.

In the emergency room, he received olanzapine and lorazepam. A urine drug screen was positive for cannabinoids. On admission, his vital signs included a blood pressure of 142/58 mmHg, a heart rate of 122 bpm, a respiratory rate of 20 breaths per minute, and a temperature of 36.1°C (97°F). Psychiatric assessment revealed increased psychomotor activity, restlessness, an irritable and labile affect, pressured speech, and a disorganized thought process. He endorsed grandiose delusions and demonstrated poor insight and judgment, consistent with acute mania. An initial diagnosis of psychosis unspecified was made, with a differential diagnosis of bipolar mania with psychosis and substance-induced psychosis.

During his hospitalization, he remained hyperactive, continued expressing grandiose delusions, and discussed involvement with gangs. He also expressed interest in obtaining a prescription for medical marijuana to manage his anxiety. He required frequent redirection for intrusive behaviors. He admitted to regularly smoking Δ8-THC (AK47), which he had purchased from an online store.

He was started on quetiapine and divalproex sodium, which were titrated to 600 mg and 1,500 mg daily, respectively. His manic symptoms gradually subsided, and he was discharged after 17 days. Outpatient treatment was initiated; however, two weeks later, he relapsed, began using alcohol, discontinued his medications, and presented to the emergency department with a recurrence of symptoms. He was readmitted, and his medications were adjusted. Due to elevated ammonia levels associated with divalproex and quetiapine, his treatment regimen was switched to lithium and olanzapine.

Following his second discharge, he has maintained adherence to treatment, abstained from cannabinoids and alcohol, and has not required further hospitalization.

The laboratory results on admission for all three cases are presented in Table 1.

**Table 1:** Laboratory results on admission for all three cases

Variable	Case 1	Case 2	Case 3	Reference range
Glucose	101	100	74	70-100 mg/dl
Blood urea nitrogen	6	6	19	6-24 mg/dl
Creatinine	1.01	1.08	0.98	0.60-1.30 mg/dl
Sodium	139	139	135	135-145 mmol/L
Potassium	4.3	3.2	4.0	3.7-5.1 mmol/L
Chloride	111	107	104	96-110 mmol/L
Bicarbonate	24.0	26.0	17.0	22.0-32.0 mmol/L
Anion gap	8	9	18	≤20 mmol/L
Calcium	9.1	8.8	8.7	8.5-10.5 mg/dl
Total protein	7.5	7.8	7.4	6.0-8.4 gm/dl
Albumin	3.9	4.1	4.1	3.5-5.0 gm/dl
Globulin	3.6	3.7	3.3	2.0-4.4 gm/dl
Aspartate aminotransferase	10	20	170	10-40 u/l
Alkaline phosphatase	66	53	58	33-138 u/l
Bilirubin, total	0.7	0.5	1.5	0.0-1.5 mg/dl
Alanine aminotransferase	20	35	99	12-78 u/l
Estimated glomerular filtration rate	76	>90	>90	≥90 mL/min/1.73 m²
Thyroid-stimulating hormone	4.211	Unavailable	1.523	0.550-4.780 UIU/ml
Alcohol, ethyl	<10	<10	<10	≤10 mg/dl
White blood cell count	9.8	8.3	12.2	4.0-12.0 k/ul
Red blood cell count	4.46	4.95	5.09	3.50-5.30 m/ul
Hemoglobin	12.8	14.7	14.5	12.0-16.0 gm/dl
Hematocrit	39.0	42.6	41.9	36.0-48.0%
Platelets	209	236	259	140-440 k/ul
Alcohol, urine	Neg screen	Neg screen	Neg screen	Neg screen
Amphetamine	Neg screen	Neg screen	Neg screen	Neg screen
Barbiturates	Neg screen	Neg screen	Neg screen	Neg screen
Opiates	Neg screen	Neg screen	Neg screen	Neg screen
Cocaine	Neg screen	Neg screen	Neg Screen	Neg screen
Benzodiazepines	Neg screen	Neg screen	Neg screen	Neg screen
Phencyclidine	Neg screen	Neg screen	Neg screen	Neg screen
Cannabinoid	Positive	Positive	Positive	Neg screen

## Discussion

With the passage of the Agricultural Improvement Act of 2018, also known as the Farm Bill, hemp - defined as *Cannabis sativa* L. and its derivatives containing no more than 0.3% Δ9-THC on a dry weight basis - was removed from the Controlled Substances Act definition of marijuana [1].

Δ8-THC is a cannabinoid that occurs naturally in cannabis plants at low concentrations but can be synthesized from hemp-derived CBD through a series of chemical reactions [2]. It is an isomer of Δ9-THC and acts on the same cannabinoid receptors [2], with Δ9-THC being the primary compound responsible for the psychoactive effects of cannabis. Given their structural similarity, Δ8-THC is believed to produce effects and side effects comparable to Δ9-THC. A study by Hollister and Gillespie found that Δ8-THC exhibited similar effects to Δ9-THC, with a relative potency ratio of 2:3 [6].

The Farm Bill facilitated the production and sale of hemp-derived products containing less than 0.3% Δ9-THC. However, since the bill did not explicitly address Δ8-THC, a perceived legal loophole has contributed to the growing popularity of Δ8-THC synthesized from hemp-derived CBD.

The adverse effects of regular or heavy Δ9-THC use are well documented, including addiction, cognitive impairment, and an increased risk of psychosis, including schizophrenia [3]. Studies have shown a positive correlation between cannabis use and psychosis risk [4]. Additionally, cannabis use in late adolescence and early adulthood has been linked to long-term negative outcomes such as lower educational attainment, reduced income, and diminished life satisfaction [5].

Public interest in Δ8-THC has surged in recent years [8,9]. Reports indicate that Google searches for Δ8-THC increased significantly from 2020 to 2021, particularly in US states with restrictions on Δ9-THC use [8,9]. It is hypothesized that Δ8-THC is being used as a legal substitute for Δ9-THC in states where marijuana remains illegal or requires medical authorization [8].

In a study by Kruger and Kruger, Δ8-THC users primarily reported positive effects, including relaxation, pain relief, and euphoria, although some experienced cognitive distortions such as altered time perception, short-term memory impairment, and difficulty concentrating [10]. Participants described the effects of Δ8-THC as milder and shorter-lasting than those of Δ9-THC [10]. Some users in the study expressed concerns about continued legal access to Δ8-THC, with many considering it a highly effective alternative to Δ9-THC with fewer side effects [10]. Another study found that more than half of the participants used Δ8-THC to self-medicate for mental health conditions and chronic pain [11].

Currently, there is limited scientific research on the short- and long-term effects of Δ8-THC use. Miller et al. reported three cases of patients requiring inpatient psychiatric admission for psychosis following Δ8-THC use [12]. Another case series described four pediatric patients who developed confusion, somnolence, seizure-like activity, tachycardia, and hypotension after exposure to Δ8-THC [13]. Additionally, a case report documented a two-year-old female who ingested Δ8-THC gummies and arrived at the hospital minimally responsive, requiring intubation [14].

Another significant concern is the presence of impurities in Δ8-THC products, often exceeding declared amounts [15]. These impurities may result from contaminants in the CBD used for synthesis, inadequate purification processes, or insufficient quality control during certification [15].

The FDA has received multiple reports of adverse events related to Δ8-THC products and has issued a safety warning to consumers, specifically cautioning against the potential use of harmful chemicals during manufacturing and the risk of production occurring in uncontrolled or unsanitary conditions [16].

## Conclusions

Δ8-THC products, due to their structural similarity and shared receptor activation with Δ9-THC, may pose similar risks, including addiction and psychosis. These risks may be heightened in individuals with preexisting psychiatric conditions and in adolescents or young adults. Given the increasing availability of Δ8-THC products and their perception as a safer, legal alternative to marijuana, clinicians should engage in open discussions with patients regarding their use. The cases presented in this report may have been influenced by additional factors, such as treatment noncompliance or underlying primary psychiatric disorders. Another potential concern is the presence of impurities and higher-than-labeled concentrations of both Δ8-THC and Δ9-THC in these products. To ensure accurate diagnosis and appropriate treatment, clinicians should obtain a thorough patient history to confirm Δ8-THC use before initiating treatment. This approach will aid in guiding clinical management, psychoeducation, and prognosis.

These findings highlight the need for stricter governmental regulations at both federal and state levels to ensure the safety and quality of Δ8-THC products. Additionally, further scientific research on Δ8-THC is essential to provide consumers with reliable information and enable healthcare providers to offer evidence-based guidance.

## References

[1] (2019). Hemp Production and the 2018 Farm Bill. https://www.fda.gov/news-events/congressional-testimony/hemp-production-and-2018-farm-bill-07252019.

[2] Tagen M, Klumpers LE (2022). Review of delta-8-tetrahydrocannabinol (Δ8-THC): comparative pharmacology with Δ9-THC. Br J Pharmacol.

[3] Volkow ND, Baler RD, Compton WM, Weiss SR (2014). Adverse health effects of marijuana use. N Engl J Med.

[4] Marconi A, Di Forti M, Lewis CM, Murray RM, Vassos E (2016). Meta-analysis of the association between the level of cannabis use and risk of psychosis. Schizophr Bull.

[5] Fergusson DM, Boden JM (2008). Cannabis use and later life outcomes. Addiction.

[6] Hollister LE, Gillespie HK (1973). Delta-8- and delta-9-tetrahydrocannabinol; comparison in man by oral and intravenous administration. Clin Pharmacol Ther.

[7] Livne O, Budney A, Borodovsky J (2022). Delta-8 THC use in US adults: sociodemographic characteristics and correlates. Addict Behav.

[8] Livingston MD, Walker A, Cannell MB, Rossheim ME (2022). Popularity of delta-8 THC on the internet across US states, 2021. Am J Public Health.

[9] Leas EC, Nobles AL, Shi Y, Hendrickson E (2022). Public interest in ∆8-Tetrahydrocannabinol (delta-8-THC) increased in US states that restricted ∆9-Tetrahydrocannabinol (delta-9-THC) use. Int J Drug Policy.

[10] Kruger JS, Kruger DJ (2022). Delta-8-THC: delta-9-THC's nicer younger sibling?. J Cannabis Res.

[11] Kruger DJ, Kruger JS (2023). Consumer experiences with delta-8-THC: medical use, pharmaceutical substitution, and comparisons with delta-9-THC. Cannabis Cannabinoid Res.

[12] Miller CR, Burk BG, Fargason RE, Birur B (2023). Delta-8-THC association with psychosis: a case report with literature review. Front Psychiatry.

[13] Shaker K, Nillas A, Ellison R, Martin K, Trecki J, Gerona R, Aldy K (2023). Delta-8-tetrahydrocannabinol exposure and confirmation in four pediatric patients. J Med Toxicol.

[14] Akpunonu P, Baum RA, Reckers A (2021). Sedation and acute encephalopathy in a pediatric patient following ingestion of delta-8-tetrahydrocannabinol gummies. Am J Case Rep.

[15] Ray CL, Bylo MP, Pescaglia J, Gawenis JA, Greenlief CM (2022). Delta-8 tetrahydrocannabinol product impurities. Molecules.

[16] 5 things to know about delta-8 tetrahydrocannabinol: delta-8 THC. https://www.fda.gov/consumers/consumer-updates/5-things-know-about-delta-8-tetrahydrocannabinol-delta-8-thc.

